# Prognostic effect of increased left ventricular wall thickness in severe aortic stenosis

**DOI:** 10.1186/s12947-020-00234-x

**Published:** 2021-01-06

**Authors:** Kyungil Park, Tae-Ho Park, Yoon-Seong Jo, Young-Rak Cho, Jong-Sung Park, Moo-Hyun Kim, Young-Dae Kim

**Affiliations:** Division of Cardiology, Department of Internal Medicine, Dong-A University Hospital, Daeshingongwon-Ro 26, Seo-gu, Busan, 602-715 Republic of Korea

**Keywords:** Aortic stenosis, Left ventricular hypertrophy, Echocardiography

## Abstract

**Background:**

It is unclear whether increased left ventricular (LV) thickness is associated with worse clinical outcomes in severe aortic stenosis (AS). The aim of this study was to determine the effect of increased LV wall thickness (LVWT) on major clinical outcomes in patients with severe AS.

**Methods and results:**

This study included 290 severe AS patients (mean age 69.4 ± 11.0 years; 136 females) between January 2008 and December 2018. For outcome assessment, the endpoint was defined as death from all causes, cardiovascular death, and the aortic valve replacement (AVR) surgery rate. During follow-up (48.7 ± 39.0 months), 157 patients had AVR, 43 patients died, and 28 patients died from cardiovascular causes. Patients with increased LVWT underwent AVR surgery much more than those without LVWT (60.0% vs. 39.0%, *p < 0.001*). Furthermore, in patients with increased LVWT, the all-cause and cardiovascular death rates were significantly lower in the AVR group than in the non-AVR group (8.8% vs. 27.3%, *p* < 0.001, 4.8%, vs. 21.0%, *p < 0.001*). Multivariate analysis revealed that increased LVWT, age, dyspnea, and AVR surgery were significantly correlated with cardiovascular death.

**Conclusions:**

In patients with severe AS, increased LVWT was associated with a higher AVR surgery rate and an increased rate of cardiovascular death independent of other well-known prognostic variates. Thus, these findings suggest that increased LVWT might be used as a potential prognostic factor in severe AS patients.

## Background

Left ventricular hypertrophy (LVH) is known as an adverse clinical factor in cardiovascular disease [1–4]. LVH in patients with aortic stenosis (AS) is characterized by increased left ventricular mass (LVM), which is the main compensatory mechanism to reduce systolic wall stress and preserve cardiac output. Although the development of LVH appears to be a beneficial adaptation in the early stage of AS, decompensation occurs in the late stage of AS due to myocardial fibrosis. LVH is common in patients with severe AS and severe LVH is associated with an increased risk of postoperative mortality after aortic valve replacement (AVR) [5]. According to previous studies, the onset of dyspnea, angina, or syncope symptoms was an indicator of impending death in patients with AS, whereas the outcome in asymptomatic AS patients was considered benign [6]. For severe AS patients, recent guidelines assigned a Class I indication for AVR to patients with symptoms, and for those without symptoms who had systolic dysfunction or another cardiac surgery [7, 8]. Progression of LVH in patients with AS may predate onset of symptoms and excessive LVH is associated with adverse outcome even in asymptomatic severe AS patients [9]. However, it is not included in the current indications of AVR [8]. Thus, we aimed to examine the association of the presence of LVH determined by preoperative echocardiography with clinical outcomes in severe AS patients.

## Methods

### Patient selection

From January 2008 to December 2018, all echocardiographic studies performed at Dong-A University Hospital were reviewed to identify severe native AS patients. A total of 290 patients were enrolled in this study. The data collection finished in December 2019 for the evaluation of AVR and death. The clinical characteristics and laboratory values of the patients were extracted from the electronic medical records. The clinical data included age, gender, height, weight, body surface area (BSA), body mass index (BMI), and blood pressure (BP). The symptoms were defined as the presence of angina, syncope, or dyspnea NYHA class ≥ II. Coronary artery disease (CAD) was defined by > 70% luminal reduction in major coronary arteries. The incidence of AVR, death from all causes, and cardiovascular death were compared between patients with increased left ventricular wall thickness (LVWT) and normal LVWT during the follow-up period. This retrospective study was approved by the Institutional Review Board of Dong-A University Hospital.

### Echocardiography

Two-dimensional and Doppler examinations were performed using a commercially available echocardiographic system (Sonos 7500 or IE33; Philips Medical Systems) equipped with a 2.5-MHz transducer. The LV end-diastolic dimension (LVEDD), LV end-systolic dimension (LVESD), interventricular septum (IVS), and posterior wall thickness (PWT) were measured, and LV ejection fraction (LVEF) was assessed using modified Simpson method. The LVM and LVM index (LVMI) were calculated from the parasternal M-mode measurements using validated formula. The left atrial (LA) volume was measured using the biplane Simpson method at end-systole from apical 4- and 2-chamber views. The LA volume index (LAVI) was determined as the LA volume divided by the body surface area [7]. Increased LVWT was defined as IVS > 1.0 cm or PWT > 1.0 cm [7]. Calculation of the relative wall thickness (RWT) with the formula (2 × PWT)/(LVEDD) permits four patterns of LVH: 1) concentric hypertrophy (increased LVM with RWT > 0.42), 2) eccentric hypertrophy (increased LVM with RWT ≤ 0.42), 3) concentric remodeling (normal LVM with RWT > 0.42), and 4) normal geometry (normal LVM with RWT ≤ 0.42) [10]. Increased LVM was defined as LVMI > 95 g/m^2^ in women and > 115 g/m^2^ in men [11]. Continuous wave-Doppler was used to assess the peak aortic velocity and the mean PG. AVA was determined using the continuity equation. Severe native AS was defined as the presence of at least one of the following criteria: peak aortic velocity > 4 m/s, mean pressure gradient (PG) > 40 mmHg, aortic valve area (AVA) < 1 cm^2^, or indexed AVA (AVAI) < 0.6 cm^2^/m^2^ [12]. Grading of the severity of AR (aortic regurgitation) was based on the ACC/AHA guidelines [12].

### Statistical analysis

Continuous variables are presented as the mean ± SD. Differences between two groups were compared with t-test. Categorical variables are presented as numbers with percentages and were compared with the chi-squared test. To find out the independent risk factors for cardiovascular death in severe AS, logistic regression analysis was performed. Variables with *p*-values of 0.2 in univariate analyses were included in multivariate analysis. A *P* < 0.05 was regarded as the statistical significance in all tests. All analyses were conducted using SPSS version 20.0 (SPSS, Chicago, IL, USA).

## Results

**Fig. 1:**
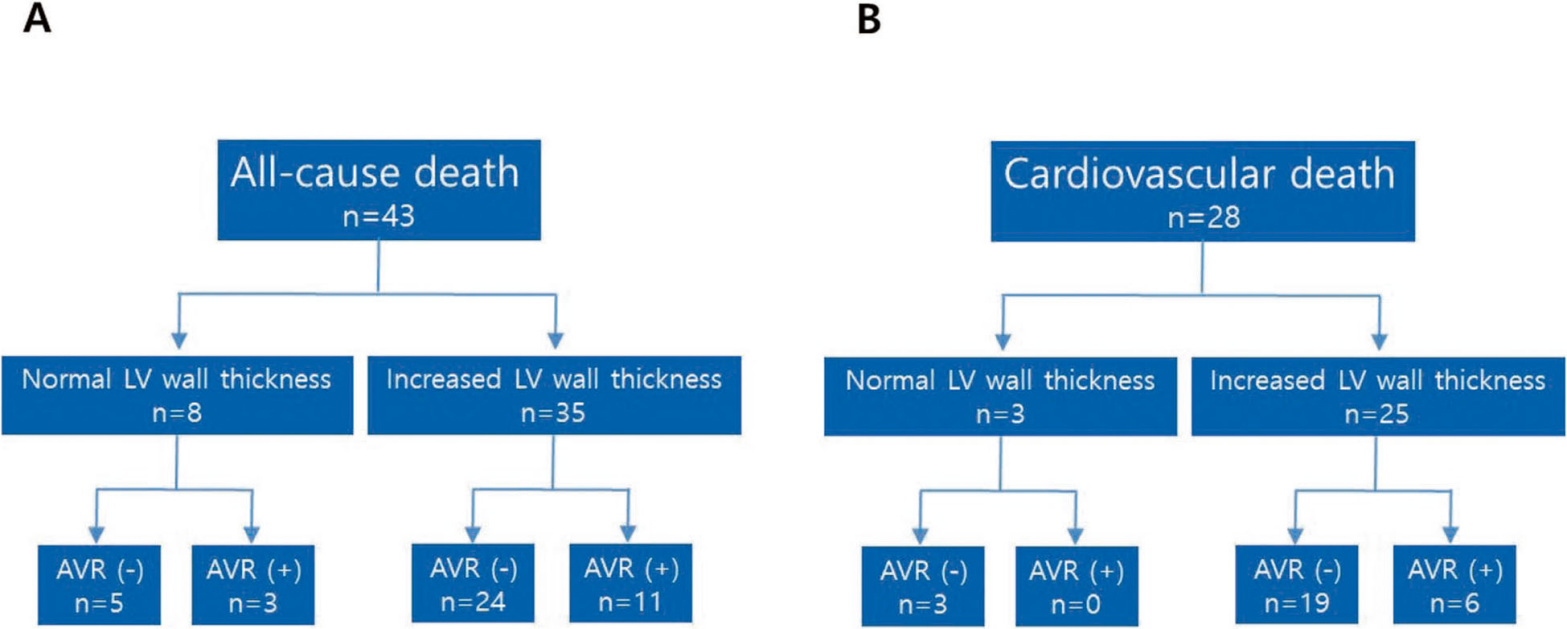
Mortality in severe AS. During a mean follow-up of 48.7 ± 39.0 months, a total of 43 patients died, 28 from cardiovascular causes. The all-cause death rate of the patients with normal LVWT was similar between the AVR and non-AVR groups (*P* = .926, **a**). However, the all-cause death rate in patients with increased LVWT was significantly lower in the AVR group (*n* = 11) than in the non-AVR group (*n* = 24) (*P* < .001, **a**). There were three cardiovascular deaths in the non-AVR group and 0 in the AVR group in patients with normal LVWT (**b**). The cardiovascular death rate in patients with increased LVWT was significantly lower in the AVR group (*n* = 6) than in the non-AVR group (*n* = 19) (*P* < .001, **b**). LVWT, left ventricular wall thickness

Compared to patients with normal LVWT, patients with increased LVWT showed higher number hypertension and dyspnea, respectively, and a higher mean NYHA class at baseline (Table 1). During follow-up (48.7 ± 39.0 months), a total of 157 patients had AVR and 43 patients died, 28 from cardiovascular causes. Thirty-two patients with normal LVWT (39.0%) underwent AVR, whereas AVR was performed in 125 patients with increased LVWT (60.0%) (*p < 0.001*). Although the all-cause death rate was not significantly different between the normal LVWT and the increased LVWT groups (9.8% vs. 16.8%, *p = 0.127*), the cardiovascular death rate was significantly higher in patients with increased LVWT than in those with normal LVWT (12.0% vs. 3.7%, *p = 0.030*, Table 1). Subgroup analysis of the death rate according to AVR surgery showed that the all-cause death rate of patients with normal LVWT was similar between the patients without and with AVR (5/50, 10.0% vs. 3/32, 9.4%, *p = 0.926*). However, the all-cause death rate in patients with increased LVWT was significantly lower in the AVR group than in the non-AVR group (11/125, 8.8% vs. 24/83, 28.9%, *p < 0.001*, Fig. 1a). Likewise, the cardiovascular death rate in the patients with increased LVWT was significantly lower in the AVR group than in the non-AVR group (6/125, 4.8% vs. 19/83, 22.9%, *p < 0.001*, Fig. 1b). Coronary angiography was done in 147 patients (50.7%). The prevalence of CAD in increased LVWT groups was not significantly different from that of normal LVWT (44.2% vs. 41.9%, *p = 0.792*).

**Table 1:** Clinical characteristics

	Normal thickness (*n* = 82)	Increased thickness (*n* = 208)	*p* value
Age (years)	69.8 ± 11.3	69.3 ± 10.9	0.707
Female, n (%)	36 (43.9%)	100 (48.1%)	0.523
Body surface area (m^2^)	1.61 ± 0.15	1.62 ± 0.16	0.497
Body mass index (kg/m^2^)	23.1 ± 3.1	23.3 ± 3.0	0.790
Hypertension, n (%)	29 (35.4%)	102 (49.3%)	0.032
Diabetes mellitus, n (%)	23 (28.0%)	63 (30.3%)	0.707
NYHA class	2.0 ± 0.9	2.3 ± 0.9	0.030
Dyspnea, n (%)	50 (61.0%)	154 (74.0%)	0.028
Angina, n (%)	26 (31.7%)	75 (36.1%)	0.484
Syncope, n (%)	3 (3.7%)	18 (8.7%)	0.139
Serum creatinine (mg/dL)	1.1 ± 0.9	1.3 ± 1.1	0.404
AVR, n (%)	32 (39.0%)	125 (60.1%)	0.001
HF hospitalization, n (%)	25 (30.5%)	61 (29.3%)	0.845
Total death, n (%)	8 (9.8%)	35 (16.8%)	0.127
Cardiovascular death, n (%)	3 (3.7%)	25 (12.0%)	0.030
Antihypertensive drugs, n (%)	40 (48.8%)	140 (67.3%)	0.003
BNP (pg/mL)	834.9 ± 1169.5	915.9 ± 1006.6	0.672

Values are mean ± SD or n (%). *NYHA* New York Heart Association; *AVR* aortic valve replacement; *HF* heart failure

### Echocardiography

**Fig. 2:**
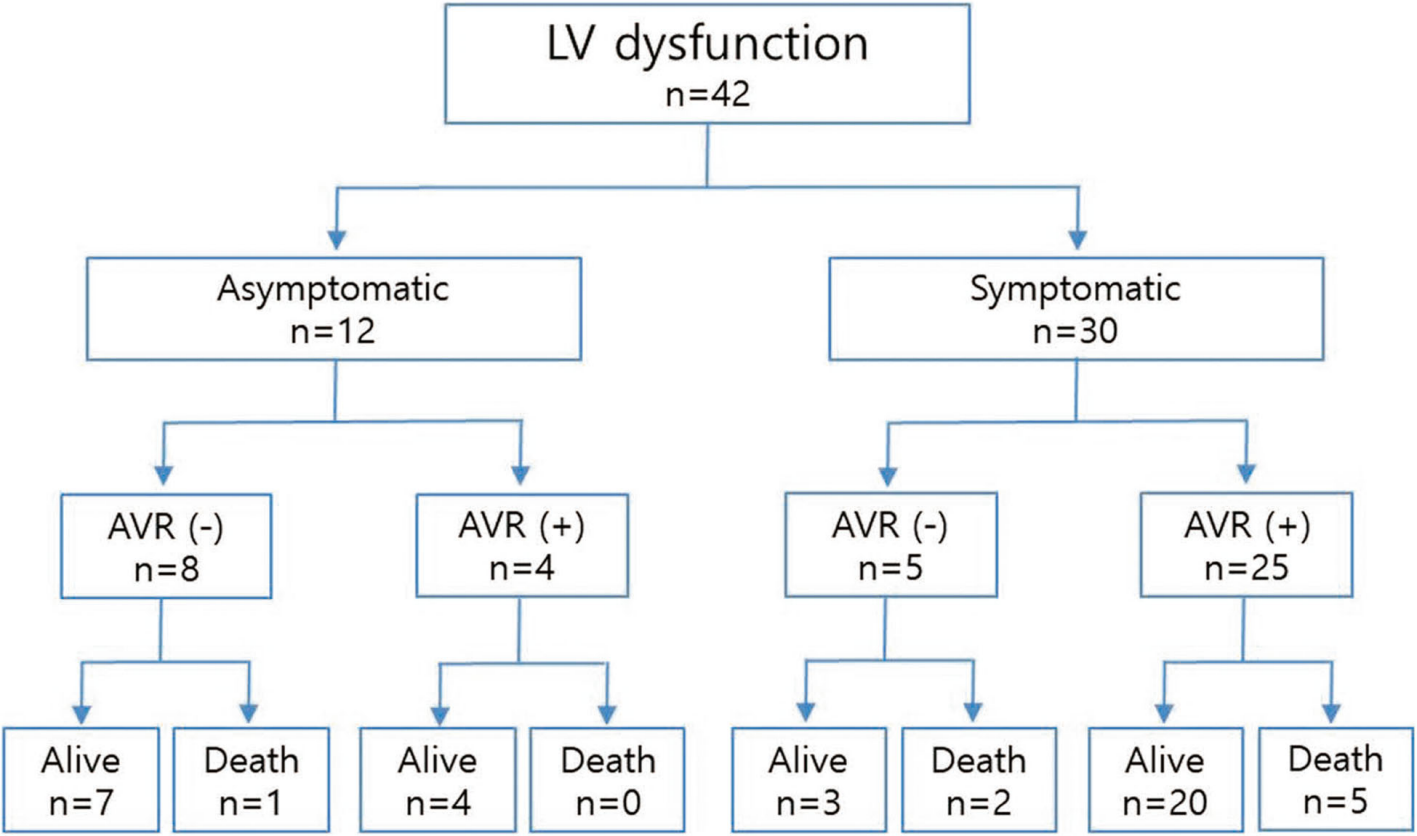
Patients with severe AS and LVEF < 50%. In our study, a total of 42 patients (14.5%) had an LVEF < 50%. During the mean follow-up of 48.7 ± 39.0 months, 29 patients (69.0%) underwent AVR surgery and 13 patients did not. Most patients with symptomatic LV dysfunction underwent AVR (83.3%), whereas only four patients with asymptomatic LV dysfunction underwent AVR and all were alive after AVR

Compared to the group with normal LVWT, the increased LVWT group showed significantly higher IVS, PWT, LVM, and LVMI and significantly lower mitral E. The AV peak pressure gradient (PG) and the mean PG were significantly higher in the group with increased LVWT than in the group with normal LVWT. AVA and AVAI were significantly smaller in the group with increased LVWT than in the group with normal LVWT (Table 2). Using the four categories of LV remodeling patterns, 177 patients (61.0%) had concentric hypertrophy, 60 patients (20.7%) had eccentric hypertrophy, 39 patients (13.4%) had a normal pattern, and 14 patients (4.8%) had concentric remodeling. The 42 patients (14.5%) included 12 patients with asymptomatic LV dysfunction (LVEF < 50%) at the time of diagnosis. AVR surgery reduced the mortality in those patients, but it was not statistically significant, irrespective of the patient symptoms (5/29, 17.2% vs. 3/13, 23.1%, *p = 0.66*, Fig. 2).

**Table 2:** Echocardiographic data

	Normal thickness (*n* = 82)	Increased thickness (*n* = 208)	*p* value
LVEDD (mm)	50.9 ± 6.5	49.6 ± 5.8	0.425
LVESD (mm)	34.2 ± 8.2	33.3 ± 8.3	0.699
LVEF (%)	57.6 ± 10.4	57.8 ± 11.3	0.786
IVS (mm)	9.1 ± 1.0	12.2 ± 1.7	< 0.001
PWT (mm)	9.3 ± 1.0	12.4 ± 1.7	< 0.001
LVM (g)	169.4 ± 41.4	241.1 ± 56.9	< 0.001
LVMI (g/m^2^)	104.8 ± 23.7	147.6 ± 34.0	< 0.001
LAV (ml)	95.7 ± 59.1	84.9 ± 33.2	0.938
LAVI (ml/g^2^)	59.9 ± 37.0	52.8 ± 21.6	0.830
Mitral E (cm/s)	112.2 ± 48.0	90.9 ± 39.1	< 0.001
Mitral E/A	1.0 ± 0.5	0.9 ± 0.6	0.220
E/e′	19.5 ± 10.7	18.1 ± 9.0	0.551
Peak velocity (m/s)	4.3 ± 0.4	4.8 ± 0.7	< 0.001
Mean PG (mmHg)	42.5 ± 9.7	55.2 ± 17.8	< 0.001
AVA (cm^2^)	0.8 ± 0.2	0.7 ± 0.2	< 0.001
AVAI (cm^2^/m^2^)	0.5 ± 0.1	0.4 ± 0.1	< 0.001
BAV, n (%)	11 (13.4%)	37 (17.8%)	0.367
Significant AR	11 (13.4%)	26 (12.5%)	0.833
Concentric LVH, n (%)	10 (12.2%)	172 (82.7%)	< 0.001
Eccentric LVH, n (%)	31 (37.8%)	28 (13.5%)	< 0.001
Concentric remodeling, n (%)	6 (7.3%)	8 (0.4%)	0.214
Normal geometry, n (%)	35 (42.7%)	0	

Values are mean ± SD. *LVEDD*, left ventricular end diastolic dimension; *LVESD* left ventricular end systolic dimension; *LVEF* left ventricular ejection fraction; *IVS* interventricular septum; *PWT* posterior wall thickness; *LVM* left ventricular mass; *LVMI* left ventricular mass index; *LAV* left atrial volume; *LAVI* left atrial volume index; *PG* pressure gradient; *AVA* aortic valve area; *AVAI* aortic valve area index; *BAV* bicuspid aortic valve; *AR* aortic regurgitation; *LVH* left ventricular hypertrophy

### Non-AVR versus AVR

Table 3 shows a comparison of the major clinical variables between the non–AVR and AVR groups. Symptoms (especially, dyspnea) were more common in the AVR group. However, LVEF, which is a major clinical indicator for AVR surgery, was not significantly different between the two groups. The age was younger and the LVWT was thicker in AVR group, both with statistical significance.

**Table 3:** A comparison of clinical variables in patients with non-AVR versus AVR groups

	non-AVR (*n* = 133)	AVR (*n* = 157)	*p* value
Symptoms, n (%)	72 (54.1%)	117 (74.5%)	< 0.001
LVEF (%)	58.7 ± 10.8	56.9 ± 11.2	0.125
Age (years)	72.6 ± 11.1	66.7 ± 10.2	< 0.001
LVWT, n (%)	83 (62.4%)	125 (79.6%)	0.001

Values are mean ± SD or n (%). *LVEF* left ventricular ejection fraction; *LV* left ventricle; *LVWT* left ventricular wall thickness

### Multivariate analysis

Table 4 shows the results of multivariate regression analysis for cardiovascular death. Multivariate regression analysis revealed that increased LVWT (OR = 4.01, 95% CI: 1.16–10.68, *p = 0.028*), age (OR = 1.05, CI: 1.04–1.15, *p = 0.034*), dyspnea (OR = 4.82, CI: 1.23–11.51, *p = 0.040*), AVR surgery (OR = 0.10, CI: 0.04–0.49, *p < 0.001*), and CAD (OR = 4.26, CI: 1.21–14.94, *p = 0.024*) were independent predictors. However, the LVEF was not a significant risk factor for cardiovascular death (*p = 0.285*).

**Table 4:** Multivariate analysis for cardiovascular death

	OR	*p* value
Increased LVWT	4.01 (1.16–10.68)	0.028
Age	1.05 (1.04–1.15)	0.034
Dyspnea	4.82 (1.23–11.51)	0.040
AVR	0.10 (0.04–0.49)	< 0.001
CAD	4.26 (1.21–14.94)	0.024
LVEF	1.96 (0.51–4.82)	0.285

*LV* left ventricle; *AVR* aortic valve replacement; *LVEF* left ventricular ejection fraction; *LVWT* left ventricular wall thickness

## Discussion

The major findings of the present study were as follows: (i) increased LVWT was associated with a higher rate of AVR surgery, (ii) In patients with increased LVWT, the all-cause and cardiovascular death rates were both significantly lower in the AVR group than in the non-AVR group, and (iii) Increased LVWT was an independent predictor for cardiovascular death in patients with severe native AS.

Although the presence of extreme LVH in severe AS indicates poor prognosis, current guidelines do not consider LVH an indication for surgical replacement in patients with AS [13]. The indications for AVR are primarily based on the presence of clinical symptoms. However, the clinical symptoms of patients with severe AS are often difficult to differentiate from the symptoms of comorbid diseases, such as lung disease or coronary artery disease, in clinical practice. A few studies of severe AS have focused on the prognostic outcome of patients with higher LV mass or LVWT. In patients with severe AS, preoperative concentric LVH was associated with increased mortality after AVR [5]. Higher LV mass was related with worse clinical outcomes after transcatheter AVR in severe symptomatic AS [14]. In a prospective study, increased LVMI was independently related with increased cardiovascular morbidity and mortality in AS patients [15]. In addition, increased cardiovascular mortality and morbidity have been reported for asymptomatic patients with severe AS and excessive LVH [8]. Thus, we believe that increased LVWT could be an important prognostic factor in severe AS, regardless of symptoms. Although LVH in severe AS is a compensatory phenomenon to reduce wall stress and maintain cardiac output [16], it eventually causes ischemia, fibrosis, and myocardial dysfunction [17, 18]. Myocardial fibrosis independently predicts risk of mortality in patients with moderate to severe AS [19].

### Increased LVWT and AVR

Because clinical decisions should be based on the risk of mortality, AVR might not a suitable endpoint in conservatively treated patients with severe AS. However, AVR is also the most important decision in the course of treatment for severe AS from a doctor’s point of view. In our study, the main causes of AVR were the development of symptoms (92/157, 58.6%) and LV dysfunction (29/157, 18.5%). Although LV hypertrophy was not considered a risk factor at the time of AVR, this retrospective study showed that patients with increased LVWT underwent AVR much more frequently than those without LVWT (60.1% vs. 39.0%, *p = 0.001*, Table 1). Furthermore, the cardiovascular death rate in patients with increased LVWT was significantly lower in the AVR group than in the non-AVR group (Fig. 2b). These results imply that LV hypertrophy may be an important risk factor in AVR decisions.

### Mortality

The multivariate regression analysis in this study showed that increased LVWT was independently correlated with cardiovascular death in patients with severe AS. In this study, 108 patients (108/208, 51.9%) with increased LVWT had no or mild symptoms. In addition, 36 patients (36/125, 28.8%) with increased LVWT who underwent AVR had no or mild symptoms. This may imply that symptom-based decisions for interventions in severe AS might miss a chance for surgery in patients (about 30%) with no significant symptoms. Thus, LVH could be used as a more sensitive marker than symptomatic criteria for AS surgery decisions. Previous studies reported that LVH was associated with an increased rate of cardiovascular events in severe AS independent of other prognostic covariates, even in asymptomatic patients [14, 15, 20, 21]. Recently, Kang reported that the incidence of cardiovascular death was significantly lower in those who underwent early AVR surgery than asymptomatic patients with very severe AS who received conservative care [22]. In our study, patients with increased LVWT had a 4.45-fold higher risk of cardiovascular death than those with normal LVWT. Interestingly, the predictors of cardiovascular death in this study were age, symptoms, AVR surgery, CAD, and increased LVWT, not LVEF. LV dysfunction is a well-known strong predictor of worse long-term survival, but our results did not agree with those of previous studies [23]. Although LV dysfunction caused by severe AS itself is a very important risk factor for cardiovascular death and a class I indicator for AVR surgery, LV dysfunction can develop from other combined valve diseases, or post-myocardial infarction. In our study, the LVEF was not significantly different between the AVR and non-AVR group. Additionally, among 42 patients with systolic dysfunction, 12 patients were asymptomatic, and only four patients (4/125, 3.2%) had AVR surgery attributable to LV dysfunction itself (Fig. 2). Among the 29 deaths in non-AVR patients, 6 deaths (2 cardiac and 4 non-cardiac) were observed even in patients with normal systolic function. These are asymptomatic but had increased LV wall thickness. This implies that LVEF would not be a perfect indicator of AVR surgery in real practice. In contrast, increased LVWT was more common in the AVR group and showed statistical significance as a variable correlated with cardiovascular death. LVH as a factor modifying the timing for AVR is not a new concept. Previous reports clearly demonstrated associations between pre-operative LVH and post-AVR mortality [24, 25]. Thus, it might be a good additional index for AVR surgery decisions in patients with severe AS, in addition to symptoms. Bicuspid aortic valve (BAV) is associated with aortic root dilation and progressive dilatation of ascending aorta was reported [26, 27]. By contrast, others demonstrated that long-term clinical outcomes and aortic root dilatation were similar between BAV and tricuspid AV (TAV) [28, 29]. We examined whether BAV can be other prognostic factors in severe AS. However, mortality of patients with BAV were similar with those of TAV patients (10.4% vs. 15.7%, *p = 0.818*). Honda et al. have reported that concomitant AR in severe AS patients had significantly worse clinical outcomes [30]. In present study, significant AR was observed in 12.7% of severe AS patients and mortality of patients with concomitant significant AR was not significantly different from those of patients without concomitant significant AR (11.6% vs. 12.4%, *p = 0.661*). The prevalence of CAD in death patients was significantly higher than that in survived patients (66.7% vs. 39.0%, *p = 0.012*). Likewise, mortality of CAD patients was significantly higher than that of patients without CAD in severe AS (25.0% vs. 9.6%, *p = 0.012*). This study had limitations. First, because this was a retrospective study, the clinical data may be incomplete. Second, the follow-up period was clearly too short and the absolute number of patients was too small to have strong statistical power. Third, LVH may be a consequence or reflection of the AS severity, not be the independent factor, because it was obtained by narrow-range analysis of LV wall thickness. Forth, we tried to incorporate LVH as an indication for AVR with classic symptoms and LV dysfunction, but this approach is incomplete comparison, because considerable patients with symptoms or LV dysfunction did not have AVR due to their very old age and poor health condition.

## Conclusions

In conclusion, increased LVWT was an independent predictor of cardiovascular death. Thus, AVR should be strongly considered in all AS patients with increased LVWT, even in asymptomatic patients.

## Data Availability

All data generated or analysed during this study are included in this published article.
